# Salivary excretion of systemically injected [^18^F]DCFPyL in prostate cancer patients undergoing PSMA scans

**DOI:** 10.3389/fonc.2024.1367962

**Published:** 2024-04-23

**Authors:** Bruna Fernandes, Jyoti Roy, Falguni Basuli, Blake M. Warner, Liza Lindenberg, Esther Mena, Steven S. Adler, Gary L. Griffiths, Peter L. Choyke, Frank I. Lin

**Affiliations:** ^1^ Molecular Imaging Branch, Center for Cancer Research, National Cancer Institute, Bethesda, MD, United States; ^2^ Chemistry and Synthesis Center, National Heart, Lung and Blood Institute, Bethesda, MD, United States; ^3^ Salivary Disorders Unit, National Institute of Dental and Craniofacial Research, National Institutes of Health, Bethesda, MD, United States; ^4^ Clinical Research Directorate, Frederick National Laboratory for Cancer Research, Frederick, MD, United States

**Keywords:** PSMA, [^18^F]-DCFPyL, salivary excretion, PET imaging, xerostomia

## Abstract

**Introduction:**

Prostate-specific membrane antigen (PSMA) is present in high amounts in salivary glands, but it is unclear whether labeled binders of PSMA are excreted in the saliva.

**Methods:**

Ten patients with prostate cancer underwent whole-body [^18^F]DCFPyL PET/CT (NCT03181867), and saliva samples were collected between 0-120 minutes post-injection. [^18^F]DCFPyL salivary excretion was measured over 120 minutes and expressed as %ID/g. Protein-associated binding was estimated by the percentage of [^18^F]DCFPyL versus parent radiotracer.

**Results:**

All PET scans of 10 patients (69 ± 8 years) with histologically confirmed prostate cancer (PSA= 2.4 ± 2.4, and Gleason Grade = 6-9) showed high uptake of [^18^F]-DCFPyL in salivary glands while 8 patients demonstrated high uptake in the saliva at 45 minutes. The intact [^18^F]-DCFPyL (98%) was also confirmed in the saliva samples at 120 min with increasing salivary radioactivity between 30-120 min.

**Conclusion:**

Systemically injected [^18^F]DCFPyL shows salivary gland uptake, an increasing amount of which is secreted in saliva over time and is not maximized by 120 minutes post-injection. Although probably insignificant for diagnostic studies, patients undergoing PSMA-targeted therapies should be aware of radioactivity in saliva.

## 1 Introduction

Prostate-specific membrane antigen (PSMA) is overexpressed in prostate cancer (PC). Studies using PSMA-binding agents, such as 2-(3-{1-carboxy-5-[(6-[^18^F]fluoro-pyridine-3-carbonyl)-amino]-pentyl}-ureido)-pentanedioic acid ([^18^F]-DCFPyL), have demonstrated high uptake and retention of this agent in salivary glands (1, 2). While salivary uptake of imaging agents has minimal clinical impact, the clinical implication for salivary gland damage is much more important when PSMA-targeting therapeutic agents are used for targeted radionuclide therapy (TRT) (3). Salivary gland damage caused by Lu-177-PSMA-617 is noted to be mostly mild and reversible, symptoms such as dry eyes and dry mouth (xerostomia) can be severe and persistent when alpha emitters such as ^225^Ac are used, with reports of patients discontinuing therapy due to intolerable xerostomia (4).

Xerostomia in PSMA-TRT is caused by high uptake of radiolabeled compounds in the salivary glands due to either salivary gland PSMA expression and/or non-specific trapping (5). It is also unclear what happens to PSMA-ligands after salivary binding. This study aimed to shed some light on the issue by investigating the salivary excretion of patients injected with [^18^F]-DCFPyL to determine the amount and form of systemically injected PSMA-targeting agents that end up in the salivary gland.

## 2 Materials and methods

### 2.1 Study design

Ten patients with histologically confirmed prostate cancer Gleason Grade 6 to 9 and prostate-specific antigen (PSA) 0.02 to 5.31, underwent a PSMA positron emission tomography-computed tomography (PET/CT) scan. A static whole-body PET/CT was performed for 45 minutes after a 2-hour post-i.v. injection of [^18^F]-DCFPyL. Saliva samples (2-3 ml) were obtained from all patients at baseline, 15, 30, 60, and 120 minutes after [^18^F]-DCFPyL injection. Samples were analyzed by radiothin-layer chromatography (radio-TLC, Fuji FLA500 series phosphorimager), and acetonitrile-extracted saliva samples were analyzed by radio-TLC in five of ten patients. All patients were followed up within 3 days of [^18^F]-DCFPyL post-injection and imaging.

### 2.2 [^18^F]DCFPyL administration and image acquisition

[^18^F]-DCFPyL was produced under GMP conditions with high purity and specific activity (SA). All patients received a systemic dose of 222 MBq ± 1.2 MBq [^18^F]-DCFPyL and a whole-body static PET/CT scan for 45 minutes on a GE Discovery MI DR scanner. A corresponding, non-diagnostic, low-dose CT was obtained for attenuation correction and anatomic localization. Transverse, sagittal, and coronal reconstructions as well as 3D rotating maximum pixel intensity projection (MIP) images were generated.

### 2.3 Measurements of saliva radioactivity

Saliva samples were collected without salivary stimulation and measured at baseline, 15, 30, 60, and 120 minutes after [^18^F]DCFPyL injection using a gamma counter (Perkin-Elmer). There were no fasting requirements prior to sample collection. Salivary [^18^F]-DCFPyL was expressed as %ID/g and mean %ID/g of [^18^F]-DCFPyL. A total of 100 µL of patient saliva (n=5) was mixed with 100 µL of acetonitrile for protein precipitation for 5 minutes at room temperature (rt). Samples were centrifuged at 14,000 rpm for 10 min and the supernatant and precipitate were counted separately in the gamma counter. A 2 μL aliquot of the parent tracer and the supernatant samples was applied in a TLC plate (silica gel HLF, Cat # P44931-2, Miles Scientific, Newark, DE) and developed with a solution of ethyl acetate:methanol:acetic acid (8:1:1). After drying, the radio-TLC plate was exposed to a phosphorimager plate (Fuji FLA500 series) for 12-18 hours, and then scanned using the Typhoon scanner (GE Healthcare). The potential radiometabolites and parent tracer were calculated using ImageQuant TL 1D version 8.2 software.

### 2.4 Statistical analysis

Statistical analysis was performed using GraphPad Prism 8. Data were first submitted to the Shapiro–Wilk test and differences between groups were analyzed using one-way ANOVA followed by Fisher’s LSD *post hoc* test (multiple groups, multiple variables). Data are shown as mean ± SD and differences between groups were considered significant when p<0.05. Correlations were analyzed by Pearson (r, parametric data) method and were considered positive and statistically significant when r>0.5 and p<0.05.

## 3 Results

### 3.1 Population

Overall, a total of 10 male patients (69 ± 8 years) with histologically confirmed prostate cancer met the criteria for the protocol between June 2017 and February 2020. Summarized patient characteristics are shown (**Table 1**). Patients presented with a mean PSA value of 2.4 ± 2.4, ranging from 0.02 to 5.31, and Gleason Grade 6 to 9. A total of 7 patients were considered to have biochemical recurrence (BCR) while 3 untreated patients were diagnosed as high-risk primary prostate cancer. No adverse events were observed.

**Table 1:** Patient information.

Patient no.	Age	PSA values (ng/mL)	Gleason Grade	Prognosis
1	68	1.66	4+5=9	BCR
2	75	1.65	3+3=6	BCR
3	73	5.31	4+4=8	High Risk Untreated
4	61	7.2	4+4=8	High Risk Untreated
5	63	0.02	3+3=6	BCR
6	67	0.75	5+4=9	BCR
7	69	1.39	3+3=6	BCR
8	75	0.5	4+5=9	BCR
9	58	4.4	4+5=9	High Risk Untreated
10	79	1.4	4+3=7	BCR

PSA: prostate-Specific Antigen; BCR: biochemical recurrence

### 3.2 PET/CT images and salivary gland uptake

**Figure 1:**
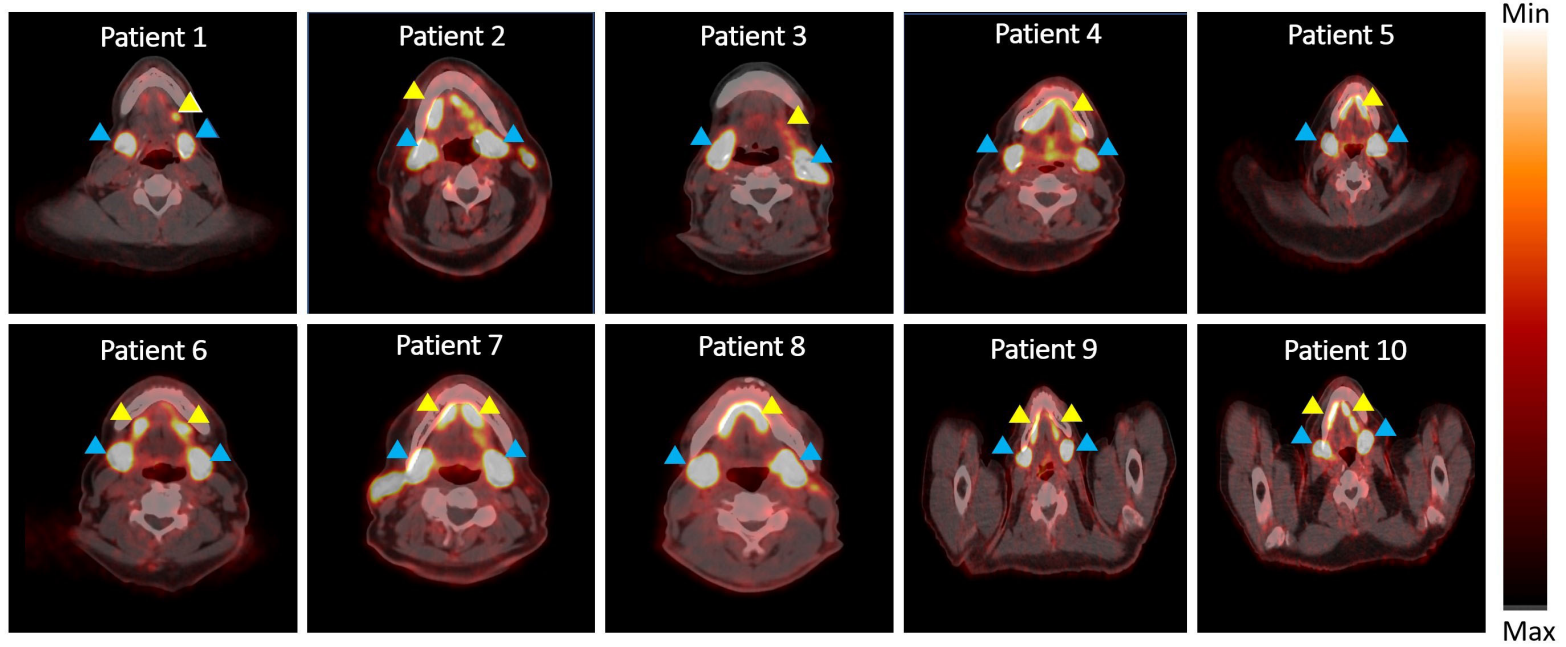
PET/CT scan showing radioactivity in salivary glands and saliva. Blue arrowhead indicates radioactivity accumulation in salivary gland and yellow arrowhead indicates radioactivity in saliva.

Representative axial PET/CT images from all ten patients are shown in **Figure 1**. PET/CT images showed high uptake of [^18^F]-DCFPyL in salivary glands (indicated by blue arrowheads) of all patients, while 8 patients demonstrated high uptake in the saliva (indicated by yellow arrowheads) and 2 patients showed medium to low saliva uptake at 45 minutes.

**Figure 2:**
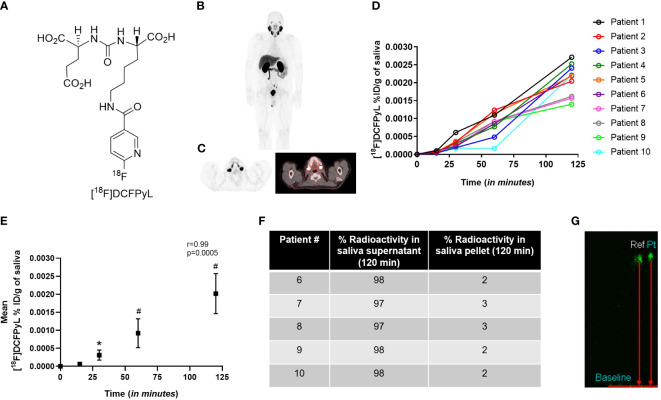
**(A)** Structure of radiolabeled PSMA ligand [^18^F]-DCFPyL, and the **(B)** representative whole body [^18^F]-DCFPyL scan. **(C)** Representative axial PET/CT scan showing [^18^F]-DCFPyL uptake in salivary glands. **(D)** %ID/g of [^18^F]-DCFPyL in saliva of patients over 120 min. **(E)** Mean %ID/g of [^18^F]-DCFPyL in saliva over 120 min. **(F)** Percentage of fluorine-18 radioactivity in saliva supernatant and pellet post-extraction of saliva in acetonitrile. **(G)** Representative TLC of reference compound [^18^F]-DCFPyL (ref) and radioactivity in saliva supernatant (pt). *p<0.05; #p<0.0001; r=Pearson correlation, TLC: thin layer chromatography.

The radiotracer [^18^F]DCFPyL (**Figure 2A**) was produced with high SA (>37 mBq/nmol) and demonstrated uptake in the salivary glands, lacrimal glands, kidneys, and liver (**Figures 2B, C**). High activity in bladder was observed due to rapid radiotracer washout and excretion from the kidneys (**Figure 2B**). The excretion of [^18^F]-DCFPyL was also confirmed in the saliva samples of all patients (**Figure 2D**). A significant difference in the tracer content in the saliva was shown at 30 min (p=0.03), 60 min (p<0.0001), and 120 min (p<0.0001) after systemic tracer injection when compared to the baseline (**Figure 2E**). A positive correlation (r=0.99, p=0.0005) was found between [^18^F]-DCFPyL uptake and advancing time-points. Of the [^18^F]-DCFPyL found in the saliva, 98% appeared in the form of intact [^18^F]-DCFPyL found in the supernatant, while approximately 2% was found bound to various proteins in the cellular pellet (**Figure 2F**). No radiometabolites were found in the saliva supernatant (**Figure 2G**).

## 4 Discussion

Human salivary glands account for approximately 35-50 grams of tissue weight in an adult male and excrete from 0.5 to 1.5 liters of saliva per day at a flow rate of approximately 0.3-0.4 mL/min. At approximate injected doses of [^18^F]-DCFPyL of 222MBq and salivary excretions of 0.002%ID/g at 2 h post-injection (**Figures 2D, E**), with 200 mL of saliva excreted in the imaging time window, it would contain 0.4% of the total injected dose (0.9 MBq). Due to similarities to other radiolabeled PSMA agents, comparable salivary activity is likely applicable in those tracers as well. While radiologically insignificant at these diagnostic doses, higher doses using beta or alpha radionuclides may increase the potential for direct damage to the mouth and other mucosal surfaces.

In a related therapeutic study in patients with metastatic salivary gland cancer doses of ^177^Lu-617 PSMA in salivary gland tumors were noted as moderate to weak, even though administered doses were the same as given to PC patients (6). Doses delivered to salivary tumors appeared not high enough to sustain therapeutic responses with this radionuclide. Similarly, both ^177^Lu-PSMA I&T and -PSMA 617 in PC patients showed the highest absorbed doses among healthy organs in lacrimal and parotid glands, but not enough to result in significant clinical side effects, nor interfere with their treatment (7).

Although only a small amount of the injected dose is found in the saliva and is likely of no concern with diagnostic doses, salivary excretion may present some tangible salivary side effect risks in patients undergoing radionuclide therapy, especially when more damaging isotopes such as alpha emitters are being used. This is especially true considering the longer physical half-life of common radioisotopes used for prostate cancer therapy, such as the 6.7 days for ^177^Lu and 9.9 days for ^225^Ac, compared to 2 hours for ^18^F used for imaging. Additionally, the small amount of radioactivity in the saliva may also have practical radio-safety implications in patients receiving treatment doses, especially to more vulnerable populations such as children and family members who may share intimate contact with treated patients. The data presented in this manuscript will help future researchers and clinicians make informed decisions about these issues.

While the data presented in this manuscript is convincing, it is important to point out that one of the limitations of the study is its small sample size, with 10 patients enrolled. Additionally, it should be noted that this study was performed with [^18^F]DCFPyL only, and the results may not generalize to other PSMA-targeting ligands that may have slightly different binding and salivary excretion kinetics. Future studies with larger samples and other PSMA-targeting molecules would be needed to confirm these findings.

## 5 Conclusion

Measurable amounts of [^18^F]-DCFPyL in saliva are to be expected in patients undergoing PET imaging with this agent, and patients should be advised accordingly.

## Data availability statement

The raw data supporting the conclusions of this article will be made available by the authors upon request.

## Ethics statement

This study involving patients was approved by the National Institute of Health Institutional Review Board. The studies were conducted in accordance with the local legislation and institutional requirements. The participants provided their written informed consent to participate in this study.

## Author contributions

BF: Data curation, Formal analysis, Writing – original draft, Writing – review & editing. JR: Data curation, Formal analysis, Methodology, Writing – original draft, Writing – review & editing. FB: Methodology, Writing – review & editing. BW: Conceptualization, Writing – review & editing. LL: Investigation, Writing – review & editing. EM: Investigation, Writing – review & editing. SA: Software, Writing – review & editing. GG: Writing – review & editing. PC: Conceptualization, Writing – review & editing. FL: Conceptualization, Formal analysis, Project administration, Supervision, Writing – review & editing.
